# To the Editors of the Medical and Physical Journal

**Published:** 1810-08

**Authors:** John Bush

**Affiliations:** Tiverton, Bath


### *To the Editors of the Medical and Phyfical Journal.*

GENTLEMEN,

IN January, 1808, Mrs. B——— having some obstruction in the urethra, attempted to relieve it by the introduction of an iron bodkin, which, slipping from her fingers, escaped into the bladder.

Soon after this accident, symptoms of inflammation of the bladder came on; a constant desire to make water, attended with almost insupportable pain, arising from the contraction of its muscular coats on the sharp ends of the bodkin.

Several weeks had elapsed from the introduction of the instrument to the bladder, before Mrs. B. became my patient, whose sufferings had much reduced her health, and had induced high symptomatic fever.

I commenced my process of cure by injecting into the bladder a pint of thin mucilage, with an ounce of olive oil, which was ordered to be retained as long as possible; and a bougie as large as the urethra would admit, was also retained till the discharge of the fluid from the bladder rendered it necessary to be withdrawn; when that took place, another injection was thrown in, and the urethra distended by a bougie as before. This plan was continued for a fortnight, care being taken to throw in an injection as soon as the last had come off, and to increase the size of the bougie every day, in order to produce mechanical distention of the urethra; at the end of this time, the irritation had ceased, and the urethra was sufficiently distended to allow the introduction of a pair of forceps, with which I extracted the bodkin. It was of iron, about two inches long; and it is worthy of remark, that it was thickly encrusted with stony matter, except about a quarter of an inch on each point, which was free from the calculous covering; this must have arisen from the bladder having contracted so firmly on the points as to have prevented the deposit from taking place.

The bodkin has been seen by Dr. Parry and several other professional gentlemen who were unacquainted with the case.

I am, &c.

JOHN BUSH.

*Tiverton, Bath, July* 16, 1810.

